# The Treatment Expectation Questionnaire (TEX-Q): Validation of a generic multidimensional scale measuring patients’ treatment expectations

**DOI:** 10.1371/journal.pone.0280472

**Published:** 2023-01-23

**Authors:** Meike C. Shedden-Mora, Jannis Alberts, Keith J. Petrie, Johannes A. C. Laferton, Pia von Blanckenburg, Sebastian Kohlmann, Yvonne Nestoriuc, Bernd Löwe

**Affiliations:** 1 Department of Psychology, Medical School Hamburg, Hamburg, Germany; 2 Department of Psychosomatic Medicine and Psychotherapy, University Medical Center Hamburg-Eppendorf, Hamburg, Germany; 3 Department of Psychological Medicine, University of Auckland, Auckland, New Zealand; 4 Department of Medicine, Health and Medical University, Potsdam, Germany; 5 Department of Clinical Psychology and Psychotherapy, Philipps-University Marburg, Marburg, Germany; 6 Department of Clinical Psychology, Helmut-Schmidt-University /University of the federal armed forces Hamburg, Hamburg, Germany; 7 Department of Systems Neuroscience, University Medical Center Hamburg-Eppendorf, Hamburg, Germany; Yokohama City University, JAPAN

## Abstract

**Background:**

Patients’ expectations, as a central mechanism behind placebo and nocebo effects, are an important predictor of health outcomes. Yet, theoretically based generic assessment tools allowing for an integrated understanding of expectations across conditions and treatments are lacking. Based on the preliminary 35-item version, this study reports the development and validation of the Treatment Expectation Questionnaire (TEX-Q), a generic, multidimensional self-report scale measuring patients’ expectations of medical and psychological treatments.

**Methods:**

The TEX-Q was developed in a validation sample of n = 251 patients undergoing different treatments using exploratory factor analyses and item analyses, as well as analysis of convergent and divergent validity. Confirmatory factor analysis was conducted in an independent sample of n = 303 patients undergoing cancer treatment. Two-weeks test-retest reliability was assessed in n = 28 psychosomatic outpatients.

**Results:**

Factor analyses revealed six theoretically founded stable subscales. The TEX-Q assesses expectations of treatment benefit, positive impact, adverse events, negative impact, process and behavioural control with a total of 15 items. Results for the subscales and the sum score indicated good internal consistency (α = .71-.92), moderate to high test-retest reliability (r = .39-.76) as well as good convergent validity with regard to other expectation measures (r = .42-.58) and divergent validity with regard to measures of generalized expectations (r < .32) and psychopathology (r < .28).

**Conclusions:**

While further validation is needed, the results suggest that the TEX-Q is a valid and reliable scale for the generic, multidimensional assessment of patients’ treatment expectations. The TEX-Q overcomes constraints of ad-hoc and disease-specific scales, while allowing to compare the impact of different expectation constructs across conditions and treatments.

## Introduction

Patients’ treatment expectations predict treatment outcome across many medical and psychological conditions [1–3]. As a central mechanism behind placebo and nocebo effects, patients’ expectations can induce relevant physiological changes that influence treatment response [4]. Positive treatment expectations predict better outcomes in many conditions and treatments including pain [5], surgery [6], musculoskeletal disorders [7], antidepressant medication [8], and psychological treatments [2]. Likewise, negative expectations can be a powerful predictor of adverse treatment outcomes such as side effects by fostering nocebo effects [9]. Overall, effect sizes for the relation between pre-treatment expectations and treatment outcome seem to be moderate. However, effect sizes vary considerably across treatments and conditions [10], and across studies within the same treatment or condition [11], pointing towards potential issues with the operationalization of expectations.

Two major aspects limit the empirical evidence for the expectations-outcome relation. On the conceptual level, the existing theoretical frameworks for treatment expectations [e.g., 12–14] have rarely been translated into the expectation measures. Thus, treatment expectations are often assessed without a clear theoretical foundation [15].

On the assessment level, most studies use disease-specific, non-validated, brief or single-item instruments, resulting in highly heterogeneous assessment. We analysed five systematic reviews investigating the assessment of expectations or their predictive value for health outcomes [2, 6, 11, 16, 17]. Out of 197 studies, 56% use single items or non-validated ad-hoc measures, which might result in unreliable data. Disease- or treatment-specific expectation measures, such as the Treatment Expectations in Chronic Pain Scale [18], are relevant for assessing unique aspects of a given disease or treatment. However, they impede the generalizability and integration of evidence across different treatments and conditions [1, 17, 19].

Moreover, our evaluation of the five systematic reviews showed that many studies assess generalized expectations, such as optimism or self-efficacy, rather than specific expectations related to treatment, such as expectations of benefit or side effects. While generalized expectations, specifically optimism, predict health outcomes, they do not provide clinically relevant information on treatment-related expectations, and sustainably improving generalized expectations can be challenging [20].

To advance the assessment of treatment expectations, a self-report scale is needed that is based on theory, focusses on outcome assessment, and captures different expectation dimensions. In order to provide the conceptual basis for such a new expectation scale, we developed the Integrative Model of Expectations in patients undergoing medical treatment [15]. The model incorporates the most relevant theories of treatment expectations, such as Kirsch’s response expectancy theory [12], Leventhal’s Common Sense Model of Illness Representation [13], and social learning and social cognitive theories [14, 21, 22]. The Integrative Model of Expectations conceptualises treatment expectations as future-directed cognitions that focus on the incidence of a specific treatment-related event or experience. Thereby, treatment expectations are distinguished from generalized expectations such as self-efficacy [21] and dispositional optimism [20]. The model further differentiates expectations directly related to the treatment [12] from expectations related to a patients’ behaviour during treatment [13, 14]. Within treatment expectations, the model distinguishes between treatment outcome-related expectations about benefit and harm of a treatment [12] and process-related expectations about the course of the treatment [10]. Regarding the outcome, expectations can focus on a continuum ranging from direct effects such as symptom improvement to broader, more distal effects such as impact on a person’s working ability. Moreover, probabilistic expectations, describing a patient’s subjective assessment regarding the likelihood of a certain outcome, can be distinguished from ideal or value-based expectation, describing wishes, hopes and fears regarding a treatment’s outcome [23, 24].

Based on the Integrative Model of Expectations, we recently developed the preliminary 35-item Treatment Expectation Questionnaire (TEX-Q), a generic, multidimensional scale assessing patients’ expectations of medical and psychological treatments [25]. The TEX-Q was developed through a systematic literature review, expert ratings to evaluate the content validity of the items, and cognitive patient interviews to evaluate the comprehensibility and acceptability [for a detailed description see 25]. With the TEX-Q, we aimed to improve the quality of evidence on the expectation-outcome connection, to allow comparability across diseases and treatments, and to comparably evaluate the impact of expectation-focused interventions. The TEX-Q was designed to be (a) generically applicable across different medical and psychological treatments, (b) multidimensional, distinguishing different expectation dimensions with potential predictive value for outcome, (c) sensitive to change in order to capture effects of expectation-focused interventions, and (d) applicable both in research and clinical practice to identify dysfunctional expectations.

The aims of this study were a) to develop a final version of the TEX-Q based on item characteristics and factorial validity, b) to determine internal consistency, test-retest reliability, as well as convergent and divergent validity, and c) to provide the final TEX-Q according to state-of-the art procedures of test translation in English and German. We hypothesized that the TEX-Q will capture the different expectations from our theoretical framework and will have sound psychometric properties. We predicted that it should show strong associations with other expectation measures, moderate associations with generalized expectation constructs, and small association with discriminant measures of psychopathology.

## Methods

Our report follows the PROMIS Instrument Development and Validation Standards [26]. Ethical approval was obtained from the Medical Chamber Hamburg, Germany (PV5790). Following open science principles, the TEX-Q will be publicly made available at https://osf.io/.

### Samples

#### Validation sample

The 35-item version of the TEX-Q was tested in consecutive patients from the following four settings to cover a range of treatment modalities: a) outpatient psychosomatic treatment at the Department of Psychosomatic Medicine and Psychotherapy, University Medical Center Hamburg-Eppendorf (UKE); b) inpatient multimodal psychosomatic treatment at the Schön Klinik Hamburg Eilbek; c) endocrine surgery at the Schön Klinik Hamburg Eilbek; and d) bariatric surgery at the Obesity Center, UKE. Inclusion criteria were planning to undergo a medical or psychological treatment and being at least 18 years old. Exclusion criteria were insufficient German language skills, inability to give informed consent, and severe psychiatric illness (e.g., psychosis), as assessed by trained study staff (psychologists at B.Sc. or M.Sc. level). Patients were recruited between August 2018 and January 2019. Eligible individuals were approached in the respective settings and completed paper-pencil questionnaires after providing written informed consent. First, they answered questions regarding sociodemographic characteristics and their condition and treatment, as well as previous treatment experiences. Second, they completed the TEX-Q, followed by the validation measures. Individuals could participate in a lottery for a 20€ voucher with a 20% chance of winning.

#### Confirmation sample

The confirmatory model fit was analysed in an independent sample of consecutive patients undergoing treatment for different types of cancer recruited at the University Clinic Giessen, Germany. Inclusion criteria included the diagnosis of cancer, planning to undergo a medical cancer treatment and being at least 18 years old. Exclusion criteria were insufficient German language skills, inability to give informed consent, and severe psychiatric illness. Patients were recruited between December 2020 and May 2021.

#### Test-retest sample

The test-retest reliability within a 2-weeks interval was analysed in a sample of patients seeking outpatient psychosomatic treatment at the Department of Psychosomatic Medicine and Psychotherapy, UKE. The same inclusion and exclusion criteria as for the validation sample applied.

### The Treatment Expectation Questionnaire (TEX-Q)–Conceptual structure and preliminary 35-item version

#### Conceptual structure

**Fig 1:**
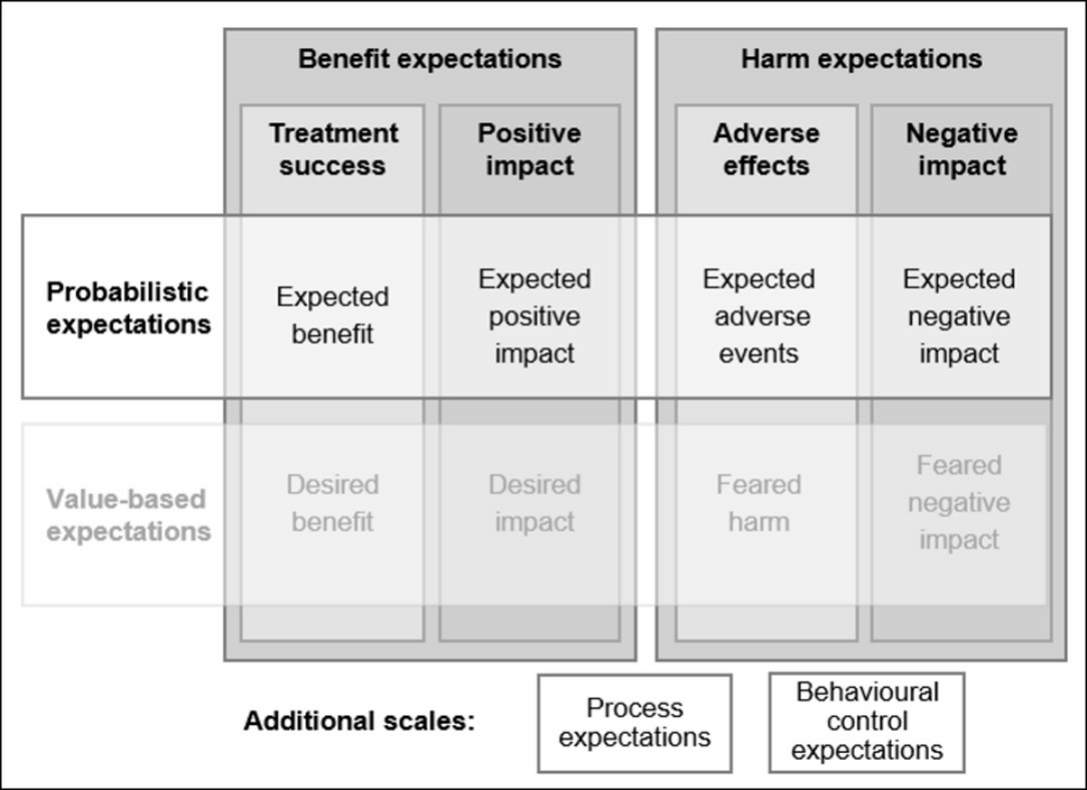
Conceptual structure of the Treatment Expectation Questionnaire (TEX-Q).

As described in the introduction, we aimed to cover relevant theoretically informed treatment-related expectation constructs that are generically measurable and potentially predictive of health outcome. The three-dimensional conceptual structure is shown in **Fig 1**. The first dimension differentiated probabilistic expectations, i.e. predictions about what is likely to happen (e.g., expecting symptom reduction), from value-based or affective expectations, i.e. hopes and fears (e.g., hoping to fully recover). This distinction was based on the theorized different predictive value of these expectation constructs [10, 23, 27]. The second dimension distinguished expectations of beneficial outcomes (e.g., treatment success) from harm expectations (e.g., side effects), based on evidence of their independent rather than unidimensional variation [21, 28]. The third dimension differentiated direct expectations (e.g., benefit or side effects) from broader, more distal expectations of treatment impact (e.g., improved quality of life), in order to cover a generic range of potential treatment outcomes [15].

This 2 x 2 x 2 structure resulted in the hypothesized subscales shown in the cells of **Fig 1**. Two additional subscales were added: *process expectations* (e.g., a straight-forward process) to cover the predictive relevance of expectations regarding the treatment process itself [13], and *behavioural control expectations* (e.g., own impact on treatment success) to capture situation-specific correlates of generalized self-efficacy [29]. Altogether, the TEX-Q included ten hypothesized subscales.

The preliminary TEX-Q contained 35 items with 3–4 items on each of the 10 subscales derived from our theoretical framework. Individuals are asked to rate their expectations, hopes and fears regarding their treatment on an 11-point Likert-scale from 0 (no change, risk, etc.) to 10 (complete relief, extreme risk, etc.).

### Measures for validation

To assess construct validity, the following validated expectation measures were administered. The Credibility/Expectancy Questionnaire (CEQ) [30] assesses treatment expectations and credibility with standardized sum scores from 3 items each using a 9-point numeric rating scale (from 1 to 9) for 4 items and a 11-point numeric rating scale (from 0% to 100%) for 2 items.

The Stanford Expectations of Treatment Scale (SETS) [28] assesses positive and negative treatment expectations with 3 items each on 7-point Likert-scales from 0 ‘not agree at all’ to 6 ‘fully agree’.

The subscale treatment control from the Brief Illness Perception Questionnaire (B-IPQ) [31] assesses treatment control expectations on an 11-point Likert-scale from 0 ‘not at all’ to 10 ‘extremely helpful’.

To assess divergent validity, the following scales were administered to distinguish the TEX-Q from generalized expectations and psychopathology.

Optimism and pessimism was measured using the Life Orientation Test (LOT-R) [32] with 3 items each on a scale ranging from 0 ‘not agree at all’ to 4 ‘fully agree’.

Generalized self-efficacy was assessed with the General Self-Efficacy Scale (GSE) [29] with 10 items on a scale ranging from 0 ‘do not agree’ to 4 ‘fully agree’.

Anxiety severity during the past 2 weeks was assessed using the Generalized Anxiety Disorder Scale (GAD-7) with 7 items on a scale ranging from 0 ‘not at all’ to 3 ‘nearly every day’ [33].

Depressive symptom severity during the past 2 weeks was assessed using the Patient Health Questionnaire (PHQ-8) with 8 items on a scale ranging from 0 ‘not at all’ to 3 ‘nearly every day’ [34].

The Positive and Negative Affect Schedule (PANAS) [35] assesses the presence of positive and negative affect using 10 items each ranging from 0 ‘little or none’ to 4 ‘extreme’.

### Translation process

The TEX-Q, originally developed in German, was translated into English according to state-of-the art procedures of test translation following the AAOS guidelines [36]. After a forward translation by two native bilingual speakers of which one was informed of the underlying concepts while the other was ‘naive’, the research team drafted a consensus translation. To check if the translation reflects the original content, this version was back-translated by two independent translators blinded to the original version. Finally, the authors (MS-M, JA, BL, KP) critically reviewed the translations and reached consensus after seeking feedback from two independent experts.

### Statistical analyses

Individuals with at least 80% available data on the TEX-Q were included in the analyses. In case of less than 80% completed items in the validation scales, individuals were excluded case-wise. Single missing values were imputed using an expectation-maximization algorithm [37].

#### Development of final version

Exploratory factor analysis (principle component analysis) using direct oblimin rotation, an eigenvalue greater than one, and the scree test to determine factor number was conducted in the validation sample. The items for the final version were selected based on factor loadings, item-total-correlations, internal consistency of the subscales, item difficulty, and content diversity. After the selection of the final items, the exploratory factor analysis was repeated.

Our à priori sample size calculation for the validation sample was based on a subject-to-item ratio of 10 to 1, the minimum number of 3 items per factor and overall sample size recommendations for test construction [38]. It suggested a sample of 300 individuals.

#### Independent confirmation of factorial structure

A confirmatory factor analysis was conducted in the confirmation sample. Global model fits were tested using the Comparative Fit Index (CFI), Tucker-Lewis Index (TLI), and the Root Mean Square Error of Approximation (RMSEA). The Lavaan package from R was used. A sample with at least 200 individuals should result in a sufficiently stable model estimate [38].

Factorial invariance was evaluated comparing psychometric properties and factor structures between the psychosomatic samples (outpatient and inpatient multimodal psychosomatic treatment) and the surgical validation samples (endocrine surgery and bariatric surgery).

#### Scale characteristics

Scale means, standard deviations, corrected item-total correlations, skewness and curtosis were calculated in the validation sample. Reliability of the scale and subscales was assessed using Cronbach’s Alpha.

#### Construct validity

Construct validity was examined in the validation sample using bivariate correlations of the TEX-Q with the validation measures. Based on theoretical assumptions, we expected high associations with the specific expectation scales (CEQ, SETS, B-IPQ) as evidence for convergent validity. In contrast, as measures for divergent validity, moderate associations with the generalized expectation measures (LOT-R, GSE), and small relations with measures of psychopathology (PHQ-8, GAD-7, PANAS) were expected.

Test-retest reliability was assessed in the test-retest sample by readministering the TEX-Q after 2 weeks. It was measured using bivariate correlations.

SPSS version 25 was used for the analyses. Level of significance was established two-sided at α = 5%.

## Results

### Sample characteristics

A total of 582 patient data sets were analysed to validate, confirm and re-test the psychometric properties of the TEX-Q.

#### Validation sample

Of 385 patients who were approached across the four settings, 263 patients (68%) participated in the study. After excluding the data sets of 12 individuals with less than 80% completed data on the TEX-Q, the final sample consisted of 251 patients. The four subsamples differed in their age, education level, illness duration, satisfaction with information and previous treatment experiences. Sample characteristics are shown in Table 1.

**Table 1:** Characteristics of the validation samples.

Variables	Total sample (n = 251)	a. Outpatient psychosomatic treatment (n = 54)	b. Inpatient multimodal psychosomatic treatment (n = 41)	c. Endocrine surgery (n = 79)	d. Bariatric surgery (n = 77)	Statistics
**Demographics**						
Age in years, M (*SD*)	43.24 (14.54)	41.42 (17.19)	34.67 (14.71)	49.13 (12.92)	43.03 (11.37)	*F*_(3.244)_ = 9.95, *p* < .001; c > a, b; b < d
Gender, female (%)	170 (68.3%)	39 (73.6%)	27 (65.9%)	59 (75.6%)	45 (58.4%)	*Χ²*_(3)_ = 6.19, *p* = .10
At least 12 years of education (%)	94 (38.2%)	26 (51.0%)	17 (41.5%)	34 (44.2%)	17 (22.1%)	*Χ²*_(3)_ = 13.35, *p* = .004
Living with someone (%)	166 (67.2%)	33 (63.5%)	29 (72.5%)	54 (69.2%)	50 (64.9%)	*Χ²*_(3)_ = 1.17, *p* = .76
**Illness / symptom characteristics**						
Duration of illness / symptoms						*F*_(3.227)_ = 11.51, *p* < .001; c > a,b,d
less than 6 months	28 (12.3%)	6 (11.5%)	3 (7.5%)	14 (18.2%)	5 (8.6%)	
6 months to 2 years	49 (21.6%)	8 (15.4%)	13 (32.5%)	26 (33.8%)	2 (3.4%)	
2–5 years	56 (24.7%)	19 (36.5%)	10 (25.0%)	18 (23.4%)	9 (15.5%)	
5–10 years	26 (11.5%)	6 (11.5%)	6 (15.0%)	7 (9.1%)	7 (12.1%)	
more than 10 years	68 (30.0%)	13 (25.0%)	8 (20.0%)	12 (15.6%)	35 (60.3%)	
How well informed about treatment? (0–6), M (*SD*)	4.45 (1.48)	3.55 (1.67)	3.88 (1.27)	5.11 (1.21)	4.68 (1.31)	*F*_(3.247)_ = 16.99, *p* < .001; a, b < c, d
Previously received same treatment for illness / symptoms (%)	38 (15.3%)	12 (23.1%)	12 (29.3%)	9 (11.4%)	5 (6.5%)	*Χ²*_(3)_ = 14.17, *p* = .003
Quality of previous treatment experience (0–6), M (*SD*)	4.02 (1.70)	4.45 (1.69)	3.67 (1.97)	4.56 (1.51)	3.58 (1.56)	*F*_(3.43)_ = 0.97, *p* = .42
Previously received any other treatment for illness / symptoms (%)	82 (33.5%)	20 (37.7%)	24 (60.0%)	15 (20.00%)	23 (29.9%)	*Χ²*_(3)_ = 19.64, *p <* .001
Overall quality of previous treatment experiences (0–6), M (*SD*)	3.40 (1.48)	3.95 (1.57)	3.33 (1.41)	3.76 (1.25)	2.86 (1.48)	*F*_(3.91)_ = 2.67, *p* = .053
**Scales**						
CEQ credibility subscale*, M (*SD*)	.00 (2.46)	-.64 (2.39)	-1.83 (3.13)	.29 (2.17)	1.12 (1.58)	*F*_(3.250)_ = 17.23, *p* < .001; a < d; b < c, d
CEQ expectancy subscale*, M (*SD*)	.00 (2.67)	-.65 (2.30)	-2.62 (3.05)	.78 (2.56)	1.05 (1.54)	*F*_(3.250)_ = 26.80, *p* < .001; a < c, d; b < a, c, d
SETS positive subscale, M (*SD*)	4.28 (1.14)	3.85 (1.11)	3.37 (1.22)	4.55 (1.08)	4.77 (.71)	*F*_(3.250)_ = 22.07, *p* < .001; a, b < c, d
SETS negative subscale, M (*SD*)	3.04 (1.81)	2.40 (1.85)	2.87 (1.84)	3.49 (1.73)	3.12 (1.73)	*F*_(3.250)_ = 4.29, *p* = .006; a < c
B-IPQ treatment control subscale, M (*SD*)	8.27 (1.72)	7.44 (1.82)	7.49 (1.95)	8.57 (1.35)	8.97 (1.47)	*F*_(3.250)_ = 13.94, *p* < .001; a, b < c, d;
LOT-R optimism, M (*SD*)	2.27 (.89)	2.00 (.76)	1.54 (.95)	2.72 (.74)	2.39 (.77)	*F*_(3.250)_ = 22.94, *p* < .001; b < a, c, d; a < c
LOT-R pessimism, M (*SD*)	1.67 (.88)	1.82 (.64)	2.28 (.98)	1.31 (.86)	1.60 (.81)	*F*_(3.250)_ = 13.36, *p* < .001; b > c, d; a > c
GSE, M (*SD*)	1.78 (.60)	1.61 (.46)	1.21 (.65)	2.02 (.46)	1.95 (.53)	*F*_(3.250)_ = 27.20, *p* < .001; b < a, c, d; a < c, d
GAD-7, M (*SD*)	9.01 (5.96)	11.06 (5.58)	12.76 (5.88)	7.82 (5.85)	6.87 (5.02)	*F*_(3.247)_ = 13.54, *p* < .001; a, b > c, d
PHQ-8, M (*SD*)	10.22 (6.27)	11.67 (5.11)	15.17 (6.23)	7.72 (6.07)	9.18 (5.51)	*F*_(3.247)_ = 17.08, *p* < .001; a > c; b > a, c, d
PANAS positive affect, M (*SD*)	1.86 (.83)	1.60 (.83)	1.37 (.83)	2.26 (.81)	1.90 (.64)	*F*_(3.250)_ = 14.63, *p* < .001; c > a, b, d; d > b
PANAS negative affect, M (*SD*)	1.70 (.94)	1.92 (.79)	2.39 (.98)	1.35 (.91)	1.54 (.83)	*F*_(3.250)_ = 14.74, *p* < .001; b > c, d; a > c

Note: *CEQ Items standardized. CEQ = Credibility/Expectancy Questionnaire; SETS = Stanford Expectations of Treatment Scale; B-IPQ = Brief Illness Perception Questionnaire; LOT-R = Life Orientation Test; GSE = General Self-Efficacy Scale; GAD-7 = Generalized Anxiety Disorder Scale; PHQ-8 = Patient Health Questionnaire; PANAS = Positive and Negative Affect Schedule

#### Confirmation sample

The confirmation sample consisted of 303 patients (36% female, mean age 62.1 years (± 13.9)) undergoing treatment for different types of cancer. The most frequent cancer types were urogenital 30.4%, dermatological 22.1%, head-neck 16.5%, and lung 13.9%. The most frequent procedures were awaiting or undergoing chemotherapy (34.7%), further diagnostic examination (26.4%) or surgery (11.9%).

#### Test-retest sample

28 patients (57.1% female, mean age 41.43 years (± 16.61)) planned for psychosomatic outpatient treatment constituted the sample for the test-retest reliability analysis.

### Initial factorial structure and further item selection

A Kaiser-Meyer-Olkin coefficient of .842 and a significant Bartlett’s test of sphericity (p < .001) indicated that the sample was adequate to perform factor analysis. The initial exploratory factor analysis in the validation sample produced a 7-factor solution, explaining 71.86% of the total variance (S1 Table). Six factors clearly represented the hypothesized conceptual structure, except the fact that probabilistic and value-based expectations loaded on the same factors for each of the four distinctions between benefit and harm expectations. One factor showed mixed factor loadings < .320, suggesting a six-factor solution.

Subsequently, based on the inability to distinguish the two hypothesized constructs in the factor analysis, we refrained from the further distinction between probabilistic and value-based expectations and only kept the probabilistic phrasings, leaving 21 items. We further reduced the number of items per subscale based on the items’ factor loadings, internal consistency of the subscales, item-total-correlations, item difficulty, and content diversity. This resulted in a 15-item version with 2–3 items per subscale.

### Final TEX-Q: Factorial structure

The resulting 15-item version of the TEX-Q was submitted to a second factor analysis in the validation sample, in which the number of extracted factors was predetermined to six. The six factors explained 81.2% of the total variance.

**Fig 2:**
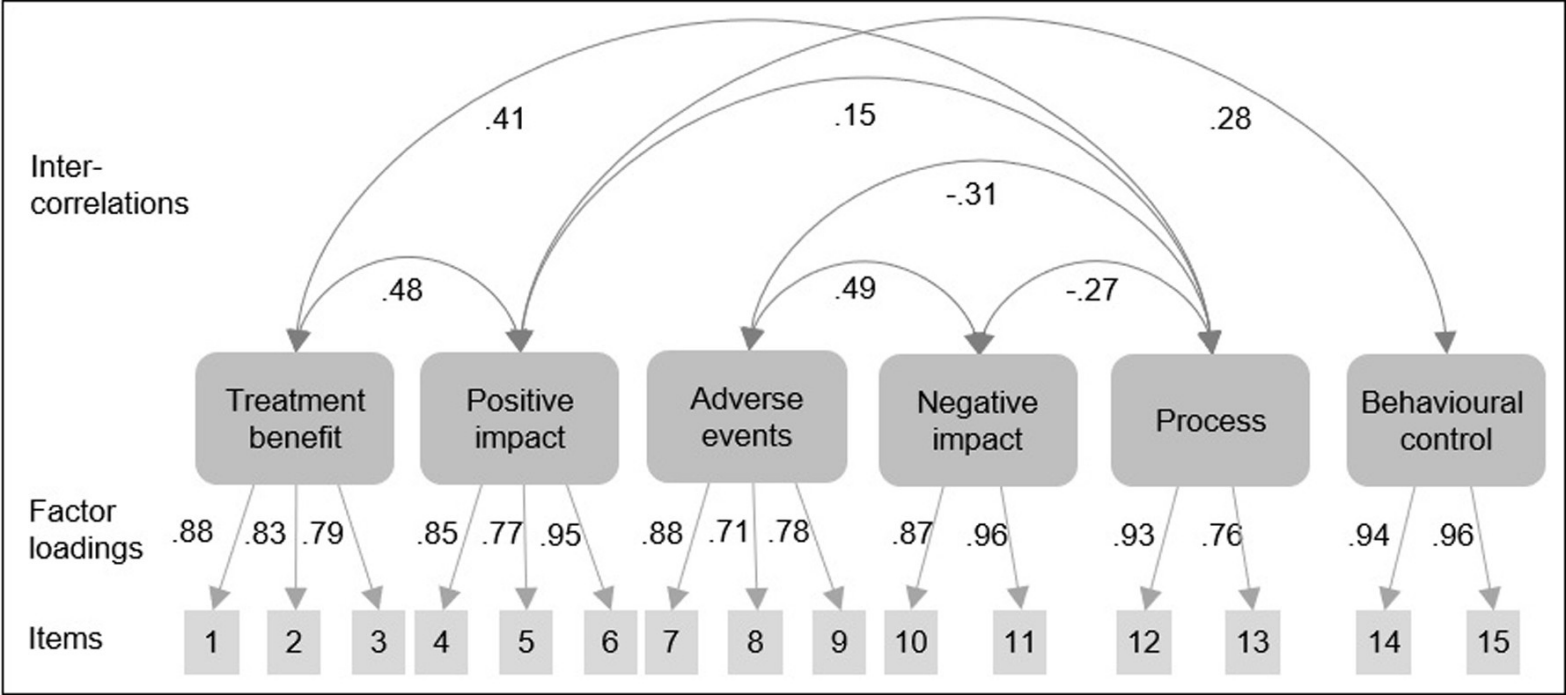
Factorial structure with factor loadings (n = 251) of the Treatment Expectation Questionnaire (TEX-Q).

As shown in **Fig 2**, the six identified subscales fully reflected the hypothesized conceptual structure, with all items loading accordingly. There were moderate correlations within the positive and negative expectation subscales. The positive and negative expectation subscales were unrelated. They correlated positively and negatively with process expectations, respectively. Expecting positive impact further correlated with higher behavioural control expectations.

#### Independent confirmation of factorial structure

Confirmatory factor analysis was used to test the identified 6-factor structure in the independent confirmation sample of 303 patients undergoing cancer treatment. Fit indices of CFI ≥ 0.95, TLI ≥ 0.95, and RMSEA < 0.08 indicate a good fit for continuous data [39]. The global model fit was good (CFI = 0.991, TLI = 0.998, RMSEA = 0.079, 90% confidence interval 0.067–0.092). All standardized factor loadings were between .722 and .994 (S2 Table). Taken together, the results support the hypothesized 6-factor model.

### Comparison between psychosomatic and surgical samples: Factorial invariance

Factorial invariance between the two psychosomatic (n = 95) and the surgical validation subsamples (n = 156) was evaluated comparing psychometric properties and factorial structures (S3 Table). The surgical treatment samples had more positive overall treatment expectations (T = -2.70, *p* = .007), higher benefit expectations (T = -6.19, *p* < .001), less negative impact expectations (T = 2.44, *p* = .015), more positive process expectations (T = -4.86, *p* < .001) and less behavioural control expectations (T = 3.31, *p* < .001).

The factor analyses of the two samples explained nearly the same amount of variance (82.1% and 81.1%), and showed a comparable factorial structure. However, the factor loadings were less unique in the psychosomatic sample. Taken together, the results suggest factorial invariance across these different treatment modalities.

### Psychometric properties

**Table 2** reports the characteristics of the six subscales and the items in the validation sample. Overall, the patients’ expectations were rather positive. The distribution was skewed for the positive impact and behavioural control scales, suggesting ceiling effects. Internal consistency of the subscales was acceptable (process) to excellent (behavioural control), and good for the overall TEX-Q (Cronbach’s α = .79). The characteristics of the other applied scales can be found in the S4 Table.

**Table 2:** Item Characteristics of the final Treatment Expectation Questionnaire (TEX-Q) in the validation sample (n = 251).

Subscale	Items	M (SD)	Corrected item total correlation*	Cronbach’s Alpha of Subscale	Cronbach’s Alpha if item deleted	Skewness (SE)	Curtosis (SE)
**Treatment benefit** (eigenvalue = 4.14, % of variance = 27.6)		**8.05 (1.67)**		**.84**		**-.98 (.15)**	**1.05 (.31)**
	1. How much relief in your symptoms do you expect from the treatment?	7.83 (2.10)	.70		.79	1.61 (.31)	.31)
	2. How much benefit do you expect from the treatment?	8.24 (1.73)	.68		.80	3.80 (.31)	(.31)
	3. How much do you expect your health will improve as a result of the treatment?	8.08 (1.91)	.75		.73	1.81 (.31)	1.81 (.31)
**Positive impact** (eigenvalue = 1.31, % of variance = 8.75)		**7.54 (2.29)**		**.86**		**-1.45 (.15)**	**2.04 (.31)**
	4. How much improvement do you expect in your ability to do your daily activities (e.g., occupation, household, social life)?	7.73 (2.56)	.76		.78	2.18 (.31)	(.31)
	5. How much do you expect the treatment will improve your quality of life?	7.82 (2.31)	.75		.80	2.24 (.31)	(.31)
	6. How much improvement do you expect in your ability to fulfil your day-to-day responsibilities (e.g., at home, at work, in the family)?	7.08 (2.89)	.71		.83	.69 (.31)	.69 (.31)
**Adverse events** (eigenvalue = 3.00, % of variance = 20.02)		**4.05 (2.16)**		**.79**		**.20 (.15)**	**-.47 (.31)**
	7. To what extent do you expect risks from the treatment?	3.51 (2.58)	.58		.77	-.70 (.31)	-.70 (.31)
	8. How much distress do you expect the treatment will cause?	4.92 (2.51)	.64		.72	-.72 (.31)	-.72 (.31)
	9. To what extent do you expect side effects or other unwanted effects from the treatment?	3.72 (2.61)	.68		.66	-.83 (.31)	-.83 (.31)
**Negative impact** (eigenvalue = 1.01, % of variance = 6.73)		**2.52 (2.41)**		**.84**		**.92 (.15)**	**.15 (.31)**
	10. How much do you expect the treatment will reduce your quality of life?	2.34 (2.45)	.73		-	1.01 (.31)	(.31)
	11. How much do you expect the treatment will limit your day-to-day responsibilities (e.g., at home, at work, in the family)?	2.71 (2.73)	.73		-	-.28 (.31)	-.28 (.31)
**Process** (eigenvalue = 0.84, % of variance = 5.61)		**7.47 (1.76)**		**.71**		**-.84 (.15)**	**.83 (.31)**
	12. To what extent do you expect the treatment procedure or process to be straight-forward?	6.96 (2.21)	.57		-	.07 (.31)	.07 (.31)
	13. To what extent do you expect to be satisfied with the treatment procedure or process?	7.98 (1.76)	.57		-	1.92 (.31)	1.92 (.31)
**Behavioural control** (eigenvalue = 1.87, % of variance = 12.49)		**7.80 (2.44)**		**.92**		**-1.52 (.15)**	**2.04 (.31)**
	14. To what extent do you expect to be responsible for the success of the treatment?	7.90 (2.57)	.85		.	1.90 (.31)	.31)
	15. To what extent do you expect your own behaviour to influence the success of the treatment?	7.71 (2.50)	.85		.	1.70 (.31)	1.70 (.31)
**TEX-Q mean** (Mean of all items after inverting items 7–11)		**7.34 (1.20)**		**.79**		**-.51 (.15)**	**.85 (.31)**

M = mean; SD = standard deviation; SE = standard error; range: 0–10, with higher score representing more positive/negative expectations.

*refers to the correlation with each subscale.

The test-retest reliability within a 2-weeks interval in the test-retest sample was high for the overall scale (Pearson’s r = .76, *p* < .001), and moderate to high for the subscales (benefit (r = .68, *p* < .001), positive impact (r = .84 p < .001), adverse events (r = .60 *p* = .001), negative impact (r = .64 *p* < .001), behavioural control (r = .73, *p* < .001)) except for lower reliability of the process subscale (r = .39, *p* = .026) (S5 Table).

### Construct validity

Regarding correlations of the TEX-Q with sociodemographic variables, older patients had more positive overall treatment expectations (r = .21, *p* < .001), higher benefit expectations (r = .18, *p* < .01), less side effect expectations (r = -.17, *p* < .01), and more positive process expectations (r = .34, *p* < .001). Patients with higher education had less side effect expectations (r = -.18, *p* < .01). In patients with previous treatment experience (n = 44), experiences and overall treatment expectations were positively correlated (r = .37, *p* < .05).

The TEX-Q mean score (with inverted items 7–11), the treatment benefit subscale, and the process subscale were moderately to strongly associated (r = .42 - .58, *p* < .001) with the other positive expectation measures, i.e., the CEQ, the SETS positive subscale and the B-IPQ treatment control subscale (Table 3). The positive impact subscale showed smaller associations (r = .28 - .32, *p* < .001). The negative subscales showed small to moderate associations with the SETS negative subscale (r = .46 and .28, *p* < .001). Behavioural control expectations were not related to the convergent validation measures.

**Table 3:** Convergent and divergent validity of the Treatment Expectation Questionnaire (TEX-Q) in the validation sample (n = 251): Pearson correlation coefficients, significance level (two tailed).

	TEX-Q total	Treat-ment benefit	Positive impact	Adverse events	Negative impact	Process	Behav-ioural control
**Convergent validity**							
Credibility/Expectancy Questionnaire (CEQ)							
CEQ credibility subscale	.47*** < .001	.46*** < .001	.28*** < .001	-.17** .008	-.19** .003	.45*** < .001	.12 .054
CEQ expectancy subscale	.51*** < .001	.58*** < .001	.32*** < .001	-.21*** < .001	-.19** .003	.42*** < .001	.07 .295
Stanford Expectations of Treatment Scale (SETS)							
SETS positive subscale	.53*** < .001	.50*** < .001	.32*** < .001	-.23*** < .001	-.28*** < .001	.45*** < .001	.08 .191
SETS negative subscale	-.24*** < .001	.04 .542	.06 .334	.46*** < .001	.28*** < .001	-.10 .117	-.07 .290
Brief Illness Perception Questionnaire (B-IPQ) treatment control subscale	.49*** < .001	.54*** < .001	.29*** < .001	-.17** .006	-.19** .002	.46*** < .001	.07 .271
**Divergent validity**							
Life Orientation Test (LOT-R)							
LOT-R optimism	.29*** < .001	.39*** < .001	.00 .992	-.20** .002	-.24*** < .001	.32*** < .001	-.07 .257
LOT-R pessimism	-.09 .168	-.20** .001	.14* .028	.10 .116	.12 .053	-.24*** < .001	.12 .066
General Self-Efficacy Scale (GSE)	.24*** < .001	.32*** < .001	.06 .339	-.11 .093	-.15* .021	.28*** < .001	-.01 .924
Generalized Anxiety Disorder Scale (GAD-7)^+^	-.12* .050	-.17** .007	.06 .327	.10 .106	.20** .001	-.15* .020	.07 .276
Patient Health Questionnaire (PHQ-8) ^+^	-.14* .023	-.24*** < .001	.10 .118	.11 .083	.17** .006	-.26***< .001	.08 .226
Positive and Negative Affect Schedule (PANAS)							
PANAS positive affect	.16* .012	.27*** < .001	-.04 .528	-.07 .246	-.08 .186	.28*** < .001	-.02 .784
PANAS negative affect	-.07 .299	-.15*.021	.15* .019	.18** .004	.16* .010	-.15* .021	.20** .001

*significant at α = .05 level (two tailed);

**significant at α = .01 level (two tailed);

***significant at α = .001 level (two tailed); ^+^ n = 248

Regarding divergent validity, the TEX-Q showed small associations (r < .32) with generalized expectation measures (LOT-R, GSE), and even smaller associations (r < .28) with measures of positive and negative affectivity (PANAS) and psychopathology (PHQ-8, GAD-7).

## Discussion

This study aimed to develop and validate the Treatment Expectation Questionnaire (TEX-Q), a generic, multidimensional measure to assess patients’ expectations of medical and psychological treatments. We expected the TEX-Q to capture the different expectation constructs from our theoretical framework and to be reliable and valid.

Factorial analyses generally confirmed our theoretical model, except the distinction between probabilistic and value-based expectations [25]. The exploratory factor revealed a six-factor model, which was mostly invariant between the psychosomatic and surgical subsamples. The structure was confirmed through confirmatory factor analysis in the independent confirmation sample. Benefit expectations were clearly distinguished from harm expectations. The fact that the benefit and harm expectation subscales were unrelated underpins the notion that positive and negative expectations are independent entities instead of one continuum [28]. These results argue for the separate use of the subscales in addition to using the TEX-Q sum score.

Regarding outcome expectations, factorial analyses distinguished direct expectations of symptom change from broader expectations of treatment impact, although factors were moderately correlated. This suggests investigating expectations regarding different outcome levels, as their relevance likely differs between conditions [15]. For instance, treatment expectations for back pain might focus on direct pain relief, while expecting improved quality of life might be more central in patients undergoing bariatric surgery. This differential predictive relevance of the TEX-Q subscales needs further evaluation.

Our inability to distinguish between probabilistic and value-based expectations adds to the mixed evidence on their proposed theoretical distinction [23, 24, 27, 40]. The analogue phrasing of our items might have diminished this differentiability. Before abandoning the distinction between probabilistic and value-based treatment expectations, more research on the role of the treatment context and the expected likelihood of a desired outcome is needed.

The psychometric properties of the TEX-Q including internal consistencies and test-retest reliabilities were satisfactory. However, ceiling effects in terms of overly positive expectations should be kept in mind, and—if confirmed in further samples—the item response options might need adaptation in future to cover the full range of expectations.

The process subscale, however, seems to be less reliable with low test-retest-reliability and only acceptable internal consistencies. Conceptually, expectations regarding the treatment process are an important aspect surrounding the individual treatment experience [10, 15]. At current stage, it seems challenging or even not possible to assess process expectations generically across treatments, as intended with the TEX-Q. In order to stimulate further research on process-related expectations, we decided to keep this subscale, but it surely needs further revision or might be excluded in a revised TEX-Q.

Regarding validity, moderate to strong associations with related expectation scales and small associations with generalized expectations and psychopathology confirm the construct validity of the TEX-Q. Importantly, the behavioural control scale and to a smaller extent the positive impact scale seem to capture different aspects of treatment expectations than previous measures and need further evaluation of construct validity. Moreover, the predictive validity of the TEX-Q needs further evaluation in future.

With the TEX-Q, we sought to overcome the limitations of the heterogeneous field of expectation research, ultimately improving our mechanistic understanding of how expectations shape outcome as an essential mechanism of placebo and nocebo effects. The TEX-Q has several advantages over previous treatment expectation measures. In contrast to treatment-specific scales [e.g., 18], it is fully generic, and–like the IPQ [31]–can be adapted to specific treatment settings. In contrast to other generic scales [e.g., 28, 30, 41, 42], it has a theory-derived multidimensional structure that allows for the comparison of the relative impact of different expectation constructs on outcome. Its modular structure allows the use of single subscales depending on research interest.

### Limitations

Several issues need consideration. Despite our effort to be aware of all imaginable treatments in the development process, the TEX-Q might not work equally well for all conditions and treatments, which means a broad validation is needed in further clinical samples. Our validation sample was limited to few conditions, although we tried to cover a range of meaningful medical and psychological treatments and settings in which placebo and nocebo effects might be of relevance [4]. While first results on factorial invariance seem promising, further evaluation of the TEX-Q and its factorial structure in a broad range of different treatments is necessary to draw more sound conclusions on its generic applicability. While the translation into English followed state of the art procedures, the psychometric evaluation is based on the German version of the TEX-Q. Thus, the psychometric properties of the English version still need to be confirmed. Moreover, the predictive validity and sensitivity to change in intervention studies need further evaluation. In particular, the usefulness in clinical settings in terms of norm values and cut-offs for dysfunctional or unrealistic expectations is yet to be studied.

On a conceptual level, our items’ phrasing is ambiguous, assessing both the magnitude of an expected change and the probability of its occurrence to a certain extent. Despite our awareness that these aspects can conceptually be distinguished [15], we chose our phrasing to capture best both aspects of magnitude and probability in the most economic and applicable way [12, 43]. Moreover, some individuals might have less pronounced expectations, and further research needs to establish whether TEX-Q is suited to capture them.

### Recommendations for using the TEX-Q

The English and German TEX-Q can be found in S1 Appendix and can be retrieved from https://osf.io/dr3gc/. Each subscale of the TEX-Q should be evaluated separately. Given our current state of evidence, we also suggest to use the overall mean score of the TEX-Q after reversing the harm expectation subscales (items 7–11), with higher values indicating more positive overall treatment expectations. When applying the TEX-Q, the general term ‘treatment’ can be replaced with the specific treatment under investigation, e.g., ‘knee surgery’. Moreover, we propose a brief assessment of treatment pre-experiences (S1 Appendix).

In order to provide a brief generic assessment tool for treatment expectations that can be included in larger clinical studies, we propose to use the *treatment benefit* (items 1–3) and *adverse events* (item 7–9) subscales separately, resulting in the brief 6-item TEX-Q-6. We expect these two subscales to capture the most relevant aspects of treatment expectations. Generally, based on the item- and scale characteristics, it seems justified to use the TEX-Q modularly, applying the subscales depending on research focus.

## Conclusions and future directions

The TEX-Q needs further evaluation and probably revision with regard to the low reliability of the process scale, distribution of the subscales, suitability of scale format, broader validation in further populations, and evaluation of prognostic validity. In addition to our tasks regarding the TEX-Q, further research on treatment expectations should shed light on the relative contribution of generalized expectation constructs such as optimism in relation to specific treatment expectations. As validated measures have shown to be better predictors of outcome than single items or ad-hoc scales [11], the TEX-Q now provides a valid basis to perform such research. Moreover, other aspects of expectations, such as implicit expectations [44] or the absence of expectations should be investigated.

To our knowledge, this is the first generic scale allowing to analyse the unique contribution of distinct expectation constructs across conditions and treatments. The TEX-Q will enable researchers in the field of placebo and nocebo mechanisms to investigate the relative impact and the predictive role of expectations for treatment outcome. Expectations are receiving increasing attention as core mechanisms of psychological interventions [45]. The TEX-Q is designed to assess the effects of expectation-focused psychological interventions [46], as in our clinical trials optimizing expectations prior to cardiac surgery [47], or targeting dysfunctional expectations to reduce side effect burden of cancer treatment [48]. It may further serve as a starting point to target dysfunctional expectations in feedback-informed therapeutic approaches [46]. Such a measure has long been called for [6, 19] and will hopefully make a valuable contribution in the expectation research field.

## Supporting information

S1 Table Exploratory factor analysis of the preliminary 35-item TEX-Q (n = 251). (DOCX)

S2 Table Confirmatory factor analysis and psychometric properties of the TEX-Q in the confirmation sample (n = 303). (DOCX)

S3 Table Factor loadings and psychometric properties of the TEX-Q in the psychosomatic and the surgical validation subsamples. (DOCX)

S4 Table Psychometric characteristics of the questionnaires applied in the validation process (n = 251). (DOCX)

S5 Table Test-retest reliability of the TEX-Q in the test-retest sample (n = 28). (DOCX)

S1 Appendix Treatment Expectation Questionnaire (TEX-Q)–English; Treatment Expectation Questionnaire (TEX-Q)–German. (DOCX)
