# Supplementary material for: The Treatment Expectation Questionnaire (TEX-Q): Validation of a generic multidimensional scale measuring patients’ treatment expectations
Source: PLoS One. 2023 Jan 23;18(1):e0280472. doi: 10.1371/journal.pone.0280472 (PMC9870103; doi:10.1371/journal.pone.0280472)
Supplement: S1 Table — (DOCX) [file pone.0280472.s001.docx]

# Supplementary Table 1: Exploratory factor analysis of the preliminary 35-item TEX-Q (n = 251)

| Items | Factor loadings | | | | | | | *M* (*SD*) |
| --- | --- | --- | --- | --- | --- | --- | --- | --- |
|  | **Factor 1** | **Factor 2** | **Factor 3** | **Factor 4** | **Factor 5** | **Factor 6** | **Factor 7** |  |
| Item 1 | .72 |  |  |  | .14 |  | -.32 | 7.83 (2.10) |
| Item 2 | .66 |  |  |  | .20 |  | -.21 | 8.24 (1.73) |
| Item 3 | .71 |  |  | -.12 | .12 |  | -.32 | 8.08 (1.91) |
| Item 4 | .74 |  |  |  | .11 |  | -.32 | 8.19 (1.91) |
| Item 15 | .86 |  |  |  |  |  | .21 | 8.49 (1.74) |
| Item 16 | .75 |  |  |  |  |  | .16 | 8.61 (1.69) |
| Item 17 | .67 |  |  | -.19 |  |  | .15 | 8.46 (1.76) |
| Item 18 | .88 |  |  |  |  |  | .17 | 8.54 (1.73) |
| Item 12 | -.14 | .63 | -.12 | -.19 |  | .12 | -.21 | 2.77 (2.75) |
| Item 13 |  | .78 |  |  | -.13 |  | -.18 | 2.34 (2.45) |
| Item 14 |  | .90 |  |  | -.13 | -.14 | -.15 | 2.71 (2.73) |
| Item 26 |  | .65 |  |  | .11 | .23 | .16 | 2.82 (2.78) |
| Item 27 |  | .66 |  |  |  | .28 | .27 | 2.68 (2.70) |
| Item 28 |  | .79 |  |  |  |  | .21 | 2.88 (2.86) |
| Item 32 |  |  | -.89 |  | .12 |  |  | 8.00 (2.23) |
| Item 33 |  |  | -.92 |  |  |  |  | 7.90 (2.57) |
| Item 34 |  |  | -.94 |  |  |  |  | 7.71 (2.50) |
| Item 35 |  |  | -.89 |  |  |  |  | 7.97 (2.34) |
| Item 5 | .24 | -.14 | -.12 | -.67 | -.11 | .14 | -.17 | 7.73 (2.56) |
| Item 6 |  |  |  | -.83 | .27 |  | -.18 | 7.51 (2,52) |
| Item 7 | .33 |  | -.10 | -.60 |  |  | -.21 | 7.82 (2.31) |
| Item 8 |  |  |  | -.86 |  | .10 | -.12 | 7.08 (2,89) |
| Item 19 | .22 |  | -.14 | -.65 | -.22 |  | .31 | 7.63 (2.74) |
| Item 20 | -.11 | .18 |  | -.84 | .14 | -.16 | .17 | 7.54 (2.69) |
| Item 21 | .37 |  | -.22 | -.50 | -.24 |  | .23 | 7.89 (2.48) |
| Item 22 |  |  | -.13 | -.74 | -.13 |  | .28 | 7.21 (2.96) |
| Item 29 |  |  |  |  | .73 | -.15 |  | 6.96 (2.21) |
| Item 30 | .21 | -.13 | -.12 |  | .68 |  | -.13 | 7.98 (1.76) |
| Item 31 |  |  | -.11 |  | .70 |  |  | 9.10 (1.45) |
| Item 9 |  | -.15 |  |  | -.12 | .85 | -.14 | 3.51 (2.58) |
| Item 10 | -.10 | .21 | -.11 |  | -.26 | .56 | -.14 | 4.92 (2.51) |
| Item 11 |  | .19 |  |  | -.12 | .69 | -.13 | 3.72 (2.61) |
| Item 23 |  |  |  |  | .17 | .87 | .17 | 3.75 (2.90) |
| Item 24 |  | .41 |  |  |  | .55 | .13 | 4.28 (2.88) |
| Item 25 |  | .24 |  |  | .13 | .71 | .18 | 3.77 (2.82) |
|  |  |  |  |  |  |  |  |  |
| Eigenvalue | 8.53 | 6.51 | 3.56 | 2.59 | 1.47 | 1.35 | 1.13 |  |
| Total variance explained | 24.38 | 18.59 | 10.17 | 7.40 | 4.21 | 3.86 | 3.23 |  |

Extraction method: principle component analysis using oblimin rotation with Kaiser normalisation.

*M* = mean; *SD* = standard deviation.
