# Supplementary material for: The Treatment Expectation Questionnaire (TEX-Q): Validation of a generic multidimensional scale measuring patients’ treatment expectations
Source: PLoS One. 2023 Jan 23;18(1):e0280472. doi: 10.1371/journal.pone.0280472 (PMC9870103; doi:10.1371/journal.pone.0280472)
Supplement: S2 Table — (DOCX) [file pone.0280472.s002.docx]

**Supplementary Table 2: Confirmatory factor analysis and psychometric properties of the TEX-Q in the confirmation sample (n = 303)**

| TEX-Q Subscales and items | Factor loadings on subscales | M (SD) | Corrected item total correlation* | Cronbach’s Alpha of Subscale | Cronbach’s Alpha if item deleted | Skewness (SE) | Curtosis (SE) |
| --- | --- | --- | --- | --- | --- | --- | --- |
| Treatment benefit | | **8.76 (1.67)** |  | **.90** |  | **-1.94 (.14)** | **4.63 (.28)** |
| 1. How much relief in your symptoms do you expect from the treatment? | 0.89 | 8.52 (2.00) | .84 |  | .82 | -1.76 (.14) | 3.44 (.28) |
| 1. How much benefit do you expect from the treatment? | 0.89 | 8.97 (1.58) | .79 |  | .87 | -2.17 (.14) | 6.05 (.28) |
| 1. How much do you expect your health will improve as a result of the treatment? | 0.97 | 8.79 (1.89) | .79 |  | .86 | -2.30 (.14) | 6.32 (.28) |
| Positive impact | | **8.27 (2.15)** |  | **.93** |  | **-1.73 (.14)** | **3.09 (.28)** |
| 1. How much improvement do you expect in your ability to do your daily activities (e.g., occupation, household, social life)? | 0.96 | 8.21 (2.40) | .88 |  | .88 | -1.72 (.14) | 2.64 (.28) |
| 1. How much do you expect the treatment will improve your quality of life? | 0.93 | 8.43 (2.17) | .83 |  | .92 | -1.92 (.14) | 3.91 (.28) |
| 1. How much improvement do you expect in your ability to fulfil your day-to-day responsibilities (e.g., at home, at work, in the family)? | 0.96 | 8.18 (2.31) | .87 |  | .89 | -1.74 (.14) | 2.92 (.28) |
| Adverse events |  | **4.30 (2.79)** |  | **.85** |  | **.16 (.14)** | **-.87 (.28)** |
| 1. To what extent do you expect risks from the treatment? | 0.79 | 4.12 (3.24) | .72 |  | .79 | .25 (.14) | -1.14 (.28) |
| 1. How much distress do you expect the treatment will cause? | 0.72 | 4.67 (3.20) | .70 |  | .80 | .06 (.14) | -1.15 (.28) |
| 1. To what extent do you expect side effects or other unwanted effects from the treatment? | 0.92 | 4.11 (3.12) | .73 |  | .78 | .25 (.14) | -1.09 (.28) |
| Negative impact |  | **3.79 (3.10)** |  | **.96** |  | **.34 (.14)** | **-1.17 (.28)** |
| 1. How much do you expect the treatment will reduce your quality of life? | 0.99 | 3.79 (3.14) | .91 |  | - | .38 (.14) | -1.11 (.28) |
| 1. How much do you expect the treatment will limit your day-to-day responsibilities (e.g., at home, at work, in the family)? | 0.95 | 3.79 (3.20) | .91 |  | - | .34 (.14) | -1.21 (.28) |
| Process |  | **8.17 (1.75)** |  | **.70** |  | **-.85 (.14)** | **.22 (.28)** |
| 1. To what extent do you expect the treatment procedure or process to be straight-forward? | 0.78 | 7.80 (2.35) | .54 |  | - | -1.18 (.14) | 1.01 (.28) |
| 1. To what extent do you expect to be satisfied with the treatment procedure or process? | 0.84 | 8.54 (1.63) | .54 |  | - | -1.19 (.14) | 1.33 (.28) |
| Behavioural control | | **7.69 (2.27)** |  | **.85** |  | **-1.18 (.14)** | **1.24 (.28)** |
| 1. To what extent do you expect to be responsible for the success of the treatment? | 0.93 | 8.03 (2.26) | .74 |  | . | -1.41 (.14) | 1.85 (.28) |
| 1. To what extent do you expect your own behaviour to influence the success of the treatment? | 0.85 | 7.35 (2.60) | .74 |  | . | -1.01 (.14) | .45 (.28) |
| TEX-Q mean (Mean of all items after inverting items 7-11) |  | **7.49 (1.47)** |  | **.88** |  | **-.42 (.14)** | **.03 (.28)** |
|  |  |  |  |  |  |  |  |

Fit indices: CFI = 0.991, TLI = 0.998, RMSEA = 0.079, 90% confidence interval 0.067 - 0.092.

*M*: mean; *SD*: standard deviation; SE: standard error; range: 0 – 10, with higher score representing more positive/negative expectations. *refers to the correlation with each subscale.
