# Supplementary material for: The Treatment Expectation Questionnaire (TEX-Q): Validation of a generic multidimensional scale measuring patients’ treatment expectations
Source: PLoS One. 2023 Jan 23;18(1):e0280472. doi: 10.1371/journal.pone.0280472 (PMC9870103; doi:10.1371/journal.pone.0280472)
Supplement: S3 Table — (DOCX) [file pone.0280472.s003.docx]

**Supplementary Table 3: Factor loadings and psychometric properties of the TEX-Q in the psychosomatic and the surgical validation subsamples**

|  | Psychosomatic treatment samples (n = 95) | | | |  | Surgical treatment samples (n = 156) | | | |
| --- | --- | --- | --- | --- | --- | --- | --- | --- | --- |
| TEX-Q Subscales and items | **Factor loadings on subscales** | **M (SD)** | **Corrected item total correlation*** | **Cronbach’s Alpha of Subscale** |  | **Factor loadings on subscales** | **M (SD)** | **Corrected item total correlation*** | **Cronbach’s Alpha of Subscale** |
| Treatment benefit |  | **7.27 (1.74)** |  | **.82** |  |  | **8.53 (1.44)** |  | **.83** |
| Item 1 | .71 | 6.84 (2.29) | .63 |  |  | .93 | 8.44 (1.73) | .68 |  |
| Item 2 | .71 | 7.73 (1.72) | .64 |  |  | .85 | 8.54 (1.67) | .69 |  |
| Item 3 | .82 | 7.24 (2.07) | .75 |  |  | .73 | 8.60 (1.61) | .68 |  |
| Positive impact |  | **7.34 (1.97)** |  | **.87** |  |  | **7.67 (2.47)** |  | **.86** |
| Item 4 | .64 | 7.45 (2.20) | .80 |  |  | .86 | 7.89 (2.75) | .75 |  |
| Item 5 | .54 | 7.45 (2.06) | .77 |  |  | .81 | 8.05 (2.42) | .74 |  |
| Item 6 | .95 | 7.11 (2.36) | .68 |  |  | .93 | 7.06 (3.18) | .73 |  |
| Adverse events |  | **4.02 (2.45)** |  | **.82** |  |  | **4.07 (1.97)** |  | **.81** |
| Item 7 | .76 | 2.82 (2.83) | .59 |  |  | .85 | 3.93 (2.33) | .65 |  |
| Item 8 | .76 | 5.62 (2.87) | .72 |  |  | .84 | 4.50 (2.17) | .66 |  |
| Item 9 | .85 | 3.63 (2.88) | .71 |  |  | .79 | 3.77 (2.43) | .66 |  |
| Negative impact |  | **2.99 (2.61)** |  | **.83** |  |  | **2.24 (2.24)** |  | **.86** |
| Item 10 | .87 | 2.65 (2.79) | .71 |  |  | .86 | 2.15 (2.20) | .75 |  |
| Item 11 | .93 | 3.34 (2.86) | .71 |  |  | .95 | 2.33 (2.58) | .75 |  |
| Process |  | **6.81 (1.98)** |  | **.69** |  |  | **7.87 (1.48)** |  | **.71** |
| Item 12 | .90 | 6.26 (2.52) | .53 |  |  | .87 | 7.38 (1.89) | .55 |  |
| Item 13 | .61 | 7.35 (1.99) | .53 |  |  | .88 | 8.37 (1.48) | .55 |  |
| Behavioural control |  | **8.44 (1.47)** |  | **.84** |  |  | **7.41 (2.81)** |  | **.93** |
| Item 14 | .90 | 8.61 (1.65) | .73 |  |  | .95 | 7.46 (2.92) | .87 |  |
| Item 15 | .94 | 8.27 (1.51) | .73 |  |  | .98 | 7.37 (2.89) | .87 |  |
|  |  |  |  |  |  |  |  |  |  |

Factor loadings of the exploratory factor analysis.

M = mean; SD = standard deviation; SE = standard error; range: 0 – 10, with higher score representing more positive/negative expectations. *refers to the correlation with each subscale.
