# Supplementary material for: The Treatment Expectation Questionnaire (TEX-Q): Validation of a generic multidimensional scale measuring patients’ treatment expectations
Source: PLoS One. 2023 Jan 23;18(1):e0280472. doi: 10.1371/journal.pone.0280472 (PMC9870103; doi:10.1371/journal.pone.0280472)
Supplement: S4 Table — (DOCX) [file pone.0280472.s004.docx]

**Supplementary Table 4: Psychometric characteristics of the questionnaires applied in the validation process (n = 251)**

| Subscale | M (SD) | Cronbach’s Alpha of Subscale | Skewness (SE) | Curtosis (SE) |
| --- | --- | --- | --- | --- |
| CEQ credibility subscale*, M (*SD*) | .00 (2.46) | .76 | -1.40 (.15) | 2.33 (.31) |
| CEQ expectancy subscale*, M (*SD*) | .00 (2.67) | .87 | -1.01 (.15) | .81 (.31) |
| SETS positive subscale, M (*SD*) | 4.28 (1.14) | .73 | -.60 (.15) | .03 (.31) |
| SETS negative subscale, M (*SD*) | 3.04 (1.81) | .87 | -.16 (.15) | -1.01 (.31) |
| B-IPQ treatment control subscale, M (*SD*) | 8.27 (1.72) | - | -1.28 (.15) | 2.01 (.31) |
| LOT-R optimism, M (*SD*) | 2.27 (.89) | .74 | -.11 (.15) | -.37 (.31) |
| LOT-R pessimism, M (*SD*) | 1.67 (.88) | .75 | .38 (.15) | .01 (.31) |
| GSE, M (*SD*) | 1.78 (.60) | .93 | -.46 (.15) | .20 (.31) |
| GAD-7, M (*SD*) ^+^ | 9.01 (5.96) | .91 | .46 (.15) | -.82 (.31) |
| PHQ-8, M (*SD*) ^+^ | 10.22 (6.27) | .90 | .41 (.15) | -.71 (.31) |
| PANAS positive affect, M (*SD*) | 1.86 (.83) | .89 | .04 (.15) | -.19 (.31) |
| PANAS negative affect, M (*SD*) | 1.70 (.94) | .89 | .37 (.15) | -.59 (.31) |

Note: *CEQ Items standardized. CEQ = Credibility/Expectancy Questionnaire; SETS = Stanford Expectations of Treatment Scale; B-IPQ = Brief Illness Perception Questionnaire; LOT-R = Life Orientation Test; GSE = General Self-Efficacy Scale; GAD-7 = Generalized Anxiety Disorder Scale; PHQ-8 = Patient Health Questionnaire; PANAS = Positive and Negative Affect Schedule; ^+^ n = 248
