# Supplementary material for: The Treatment Expectation Questionnaire (TEX-Q): Validation of a generic multidimensional scale measuring patients’ treatment expectations
Source: PLoS One. 2023 Jan 23;18(1):e0280472. doi: 10.1371/journal.pone.0280472 (PMC9870103; doi:10.1371/journal.pone.0280472)
Supplement: S5 Table — (DOCX) [file pone.0280472.s005.docx]

**Supplementary Table 5: Test-retest reliability of the TEX-Q in the test-retest sample (n=28)**

| **Subscale TEX-Q** | ***N*** | **T0 M (SD)** | **T1 M (SD)** | ***r*** | ***p*** | ***ICC*** | ***p*** |
| --- | --- | --- | --- | --- | --- | --- | --- |
| Treatment benefit | 27 | 6.80 (2.17) | 6.62 (1.88) | .68** | < .001 | .67 | < .001 |
| Positive impact | 25 | 6.85 (2.42) | 7.09 (2.00) | .84** | < .001 | .82 | < .001 |
| Adverse events | 26 | 3.35 (2.06) | 3.37 (2.38) | .60** | .001 | .59 | < .001 |
| Negative impact | 26 | 2.96 (2.12) | 3.31 (2.98) | .64** | < .001 | .61 | < .001 |
| Process | 26 | 6.48 (1.34) | 7.23 (1.33) | .39* | .026 | .39 | .024 |
| Behavioural control | 26 | 7.88 (1.94) | 8.35 (1.71) | .73** | < .001 | .72 | < .001 |
| TEX-Q mean* | 27 | 6.98 (1.26) | 7.05 (1.32) | .76** | < .001 | .76 | < .001 |

*M*: mean. *SD*: standard deviation. *r*: pearson correlation coefficients. ICC: intraclass coefficient.

*Mean of all items after inverting items 7-11
