# Supplementary material for: The Treatment Expectation Questionnaire (TEX-Q): Validation of a generic multidimensional scale measuring patients’ treatment expectations
Source: PLoS One. 2023 Jan 23;18(1):e0280472. doi: 10.1371/journal.pone.0280472 (PMC9870103; doi:10.1371/journal.pone.0280472)
Supplement: S1 Appendix — (DOCX) [file pone.0280472.s006.docx]

**S4 Appendix**

**Treatment Expectation Questionnaire (TEX-Q) – English**

**Your expectations of the treatment**

The questions below are about what you expect from your upcoming medical or psychological treatment *[can be individualized for the investigated treatment, e.g., your surgery].*

My planned treatment: ____________________________________________

For the following statements, please refer to the treatment/procedure mentioned above.

| The following section is about your **expectations** regarding your treatment. Please try to assess them as realistically as possible. | | | | | | | | | | | | | | | | | | | | | |
| --- | --- | --- | --- | --- | --- | --- | --- | --- | --- | --- | --- | --- | --- | --- | --- | --- | --- | --- | --- | --- | --- |
| 1. **How much relief in your symptoms do you expect from the treatment?** | | | | | | | | | | | | | | | | | | | | | |
| no relief | 0 | | 1 | | 2 | | 3 | | 4 | | 5 | | 6 | | 7 | | 8 | | 9 | 10 | complete relief |
| 1. **How much benefit do you expect from the treatment?** | | | | | | | | | | | | | | | | | | | | | |
| no benefit | 0 | | 1 | | 2 | | 3 | | 4 | | 5 | | 6 | | 7 | | 8 | | 9 | 10 | maximum benefit |
| 1. **How much do you expect your health will improve as a result of the treatment?** | | | | | | | | | | | | | | | | | | | | | |
| no improvement | 0 | | 1 | | 2 | | 3 | | 4 | | 5 | | 6 | | 7 | | 8 | | 9 | 10 | complete improvement |
| 1. **How much improvement do you expect in** **your ability to do your daily activities  (e.g., occupation, household, social life)?** | | | | | | | | | | | | | | | | | | | | | |
| no improvement | 0 | | 1 | | 2 | | 3 | | 4 | | 5 | | 6 | | 7 | | 8 | | 9 | 10 | complete improvement |
| 1. **How much do you expect the treatment will improve your quality of life?** | | | | | | | | | | | | | | | | | | | | | |
| no improvement | 0 | | 1 | | 2 | | 3 | | 4 | | 5 | | 6 | | 7 | | 8 | | 9 | 10 | complete improvement |
| 1. **How much improvement do you expect in** **your ability to fulfil your day-to-day responsibilities (e.g., at home, at work, in the family)?** | | | | | | | | | | | | | | | | | | | | | |
| no improvement | 0 | | 1 | | 2 | | 3 | | 4 | | 5 | | 6 | | 7 | | 8 | | 9 | 10 | complete improvement |
| 1. **To what extent do you expect risks from the treatment?** | | | | | | | | | | | | | | | | | | | | | |
| no risk | 0 | | 1 | | 2 | | 3 | | 4 | | 5 | | 6 | | 7 | | 8 | | 9 | 10 | extreme risk |
| 1. **How much distress do you expect the treatment will cause?** | | | | | | | | | | | | | | | | | | | | | |
| no distress | 0 | | 1 | | 2 | | 3 | | 4 | | 5 | | 6 | | 7 | | 8 | | 9 | 10 | extreme distress |
| 1. **To what extent do you expect side effects or other unwanted effects from the treatment?** | | | | | | | | | | | | | | | | | | | | | |
| not unwanted effects | 0 | 1 | | 2 | | 3 | | 4 | | 5 | | 6 | | 7 | | 8 | | 9 | | 10 | Extreme unwanted effects |
| 1. **How much do you expect the treatment will reduce your quality of life?** | | | | | | | | | | | | | | | | | | | | | |
| not at all | 0 | | 1 | | 2 | | 3 | | 4 | | 5 | | 6 | | 7 | | 8 | | 9 | 10 | extremely |
| 1. **How much do you expect the treatment will limit your day-to-day responsibilities  (e.g., at home, at work, in the family)?** | | | | | | | | | | | | | | | | | | | | | |
| not at all | 0 | | 1 | | 2 | | 3 | | 4 | | 5 | | 6 | | 7 | | 8 | | 9 | 10 | extremely |
| The following section is about your expectations regarding the **course** of your treatment. | | | | | | | | | | | | | | | | | | | | | |
| 1. **To what extent do you expect the treatment procedure or process to be straight-forward?** | | | | | | | | | | | | | | | | | | | | | |
| not at all | 0 | | 1 | | 2 | | 3 | | 4 | | 5 | | 6 | | 7 | | 8 | | 9 | 10 | completely |
| 1. **To what extent do you expect to be satisfied** **with the treatment procedure or process?** | | | | | | | | | | | | | | | | | | | | | |
| not at all | 0 | | 1 | | 2 | | 3 | | 4 | | 5 | | 6 | | 7 | | 8 | | 9 | 10 | extremely |
| 1. **To what extent do you expect to be responsible for the success of the treatment?** | | | | | | | | | | | | | | | | | | | | | |
| not at all | 0 | | 1 | | 2 | | 3 | | 4 | | 5 | | 6 | | 7 | | 8 | | 9 | 10 | extremely |
| 1. **To what extent do you expect your own behaviour to influence the success of the treatment?** | | | | | | | | | | | | | | | | | | | | | |
| no influence | 0 | | 1 | | 2 | | 3 | | 4 | | 5 | | 6 | | 7 | | 8 | | 9 | 10 | extremely |

**Additional items: Previous treatment experiences**

1. **Have you received your upcoming treatment before?** 0  no 1 yes

If yes, please state the date:

_____ / _________ (month / year)

1. **If yes, how do you rate your previous experience with the treatment?**

| extremely negative | | |  | |  | |  | | neutral | |  | |  | |  | | extremely positive | |
| --- | --- | --- | --- | --- | --- | --- | --- | --- | --- | --- | --- | --- | --- | --- | --- | --- | --- | --- |
| 0 | 1 | 2 | | 3 | | 4 | | 5 | | 6 | | 7 | | 8 | | 9 | | 10 |

1. **If yes, how much improvement have you experienced with your previous treatment?**

| No  improvement | | |  | |  | |  | |  | |  | |  | | complete improvement | |
| --- | --- | --- | --- | --- | --- | --- | --- | --- | --- | --- | --- | --- | --- | --- | --- | --- |
| 0 | 1 | 2 | | 3 | | 4 | | 5 | | 6 | | 7 | | 8 | 9 | 10 |

1. **How well informed do you feel about your upcoming treatment?**

| Extremely poorly informed | | |  | |  | |  | |  | |  | |  | |  | | Perfectly informed | |
| --- | --- | --- | --- | --- | --- | --- | --- | --- | --- | --- | --- | --- | --- | --- | --- | --- | --- | --- |
| 0 | 1 | 2 | | 3 | | 4 | | 5 | | 6 | | 7 | | 8 | | 9 | | 10 |

**Treatment Expectation Questionnaire (TEX-Q) – German**

**Ihre Erwartungen an die Behandlung**

Im Folgenden geht es darum, was Sie persönlich über Ihre bevorstehende medizinische oder psychotherapeutische Behandlung denken *[Individualisierung möglich, z.B. Ihre bevorstehende Operation]*.

Meine geplante Behandlung: ________________________________________________

Bitte beziehen Sie sich bei den folgenden Angaben auf die genannte Behandlung.

| Im folgenden Abschnitt geht es darum, welche **Erwartungen** Sie an Ihre Behandlung haben. Versuchen Sie hier bitte diese möglichst **realistisch** einzuschätzen. | | | | | | | | | | | | |
| --- | --- | --- | --- | --- | --- | --- | --- | --- | --- | --- | --- | --- |
| 1. **Wie viel Linderung Ihrer Symptome erwarten Sie?** | | | | | | | | | | | | |
| keine Linderung | 0 | 1 | 2 | 3 | 4 | 5 | 6 | 7 | 8 | 9 | 10 | größte vorstellbare Linderung |
| 1. **Wie viel Nutzen erwarten Sie von der Behandlung?** | | | | | | | | | | | | |
| keinen Nutzen | 0 | 1 | 2 | 3 | 4 | 5 | 6 | 7 | 8 | 9 | 10 | größter vorstellbarer Nutzen |
| 1. **Wie viel Verbesserung Ihrer Gesundheit erwarten Sie?** | | | | | | | | | | | | |
| keine Verbesserung | 0 | 1 | 2 | 3 | 4 | 5 | 6 | 7 | 8 | 9 | 10 | größte vorstellbare Verbesserung |
| 1. **Wie viel Verbesserung erwarten Sie bezüglich Ihrer Alltagsaktivitäten  (z.B. Beruf, Haushalt, Sozialleben)?** | | | | | | | | | | | | |
| keine Verbesserung | 0 | 1 | 2 | 3 | 4 | 5 | 6 | 7 | 8 | 9 | 10 | größte vorstellbare Verbesserung |
| 1. **Wie viel Verbesserung Ihrer Lebensqualität erwarten Sie?** | | | | | | | | | | | | |
| keine Verbesserung | 0 | 1 | 2 | 3 | 4 | 5 | 6 | 7 | 8 | 9 | 10 | größte vorstellbare Verbesserung |
| 1. **Wie viel Verbesserung erwarten Sie bezüglich Ihrer Verpflichtungen  (z.B. häuslich, beruflich, familiär)?** | | | | | | | | | | | | |
| keine Verbesserung | 0 | 1 | 2 | 3 | 4 | 5 | 6 | 7 | 8 | 9 | 10 | größte vorstellbare Verbesserung |
| 1. **Welches Ausmaß an Risiken erwarten Sie durch die Behandlung?** | | | | | | | | | | | | |
| keine  Risiken | 0 | 1 | 2 | 3 | 4 | 5 | 6 | 7 | 8 | 9 | 10 | größte vorstellbare Risiken |
| 1. **Wie viel Belastung erwarten Sie durch die Behandlung?** | | | | | | | | | | | | |
| keine Belastung | 0 | 1 | 2 | 3 | 4 | 5 | 6 | 7 | 8 | 9 | 10 | größte vorstellbare Belastung |
| 1. **In welchem Ausmaß erwarten Sie Nebenwirkungen oder andere unerwünschte Wirkungen?** | | | | | | | | | | | | |
| keine unerwünschten Wirkungen | 0 | 1 | 2 | 3 | 4 | 5 | 6 | 7 | 8 | 9 | 10 | größte vorstellbare unerwünschte Wirkungen |
| 1. **Wie viel Einschränkung in Bezug auf Ihre Lebensqualität erwarten Sie?** | | | | | | | | | | | | |
| keine Einschränkung | 0 | 1 | 2 | 3 | 4 | 5 | 6 | 7 | 8 | 9 | 10 | größte vorstellbare Einschränkung |
| 1. **Wie viel Einschränkung erwarten Sie bezüglich Ihrer Verpflichtungen (z.B. beruflich, häuslich, familiär)?** | | | | | | | | | | | | |
| keine Einschränkung | 0 | 1 | 2 | 3 | 4 | 5 | 6 | 7 | 8 | 9 | 10 | größte vorstellbare Einschränkung |
| Im Folgenden geht es darum, was Sie vom **Verlauf** ihrer Behandlung erwarten. | | | | | | | | | | | | |
| 1. **In welchem Ausmaß erwarten Sie einen angenehmen Behandlungsverlauf?** | | | | | | | | | | | | |
| nicht  angenehm | 0 | 1 | 2 | 3 | 4 | 5 | 6 | 7 | 8 | 9 | 10 | angenehmster vorstellbarer Verlauf |
| 1. **In welchem Ausmaß erwarten Sie, mit dem Ablauf der Behandlung zufrieden zu sein?** | | | | | | | | | | | | |
| nicht zufrieden | 0 | 1 | 2 | 3 | 4 | 5 | 6 | 7 | 8 | 9 | 10 | größte vorstellbare Zufriedenheit |
| 1. **Wie stark erwarten Sie, selbst für den Behandlungserfolg mitverantwortlich zu sein?** | | | | | | | | | | | | |
| keine Verantwortung | 0 | 1 | 2 | 3 | 4 | 5 | 6 | 7 | 8 | 9 | 10 | größte vorstellbare Verantwortung |
| 1. **Wie stark erwarten Sie, durch Ihr Verhalten auf den Behandlungserfolg Einfluss nehmen zu können?** | | | | | | | | | | | | |
| kein Einfluss | 0 | 1 | 2 | 3 | 4 | 5 | 6 | 7 | 8 | 9 | 10 | größter vorstellbarer Einfluss |

**Zusatzitems Vorerfahrungen**

1. **Haben Sie diese Behandlung schon einmal erhalten?** 0  nein 1 ja

Wenn ja, wann war das?

_____ / _________ (Monat / Jahr)

1. **Wenn ja, wie würden Sie Ihre bisherigen Erfahrungen mit dieser Behandlung bewerten?**

| sehr  negativ | |  | |  | |  | | neutral | | |  | |  | |  | | sehr positiv | |
| --- | --- | --- | --- | --- | --- | --- | --- | --- | --- | --- | --- | --- | --- | --- | --- | --- | --- | --- |
| 0 | 1 | | 2 | | 3 | | 4 | | 5 | 6 | | 7 | | 8 | | 9 | | 10 |

1. **Wenn ja, wieviel Verbesserung haben Sie durch Ihre Behandlung erlebt?**

| keine Verbesserung | | |  | |  | |  | |  | |  | | größte vorstellbare Verbesserung | | | |
| --- | --- | --- | --- | --- | --- | --- | --- | --- | --- | --- | --- | --- | --- | --- | --- | --- |
| 0 | 1 | 2 | | 3 | | 4 | | 5 | | 6 | | 7 | | 8 | 9 | 10 |

1. **Wie gut fühlen Sie sich über Ihre aktuelle Behandlung informiert?**

| Überhaupt nicht gut informiert | | |  | |  | |  | |  | |  | |  | |  | | sehr gut informiert | |
| --- | --- | --- | --- | --- | --- | --- | --- | --- | --- | --- | --- | --- | --- | --- | --- | --- | --- | --- |
| 0 | 1 | 2 | | 3 | | 4 | | 5 | | 6 | | 7 | | 8 | | 9 | | 10 |
